# Editorial: Novel sociological methods and practices of engagement across disability communities

**DOI:** 10.3389/fsoc.2025.1710857

**Published:** 2025-10-09

**Authors:** Danielle Landry, Temba Middelmann, Robel Abay, Hannah Morgan, Karen Soldatić

**Affiliations:** ^1^Canada Excellence Research Chair (CERC) Health Equity and Community Wellbeing, Toronto Metropolitan University, Toronto, ON, Canada; ^2^Independent Researcher, Toronto, ON, Canada; ^3^Alice Salomon University of Applied Sciences Berlin, Berlin, Germany; ^4^School of Sociology and Social Policy, University of Leeds, Leeds, United Kingdom

**Keywords:** disability, wellbeing, community engagement, qualitative research methods, disability studies, Mad studies, participatory approaches, ableism

We intentionally chose a magnified photograph of a broken bone as the featured image for this Research Topic. We take up this image metaphorically, rather than as a direct reference to orthopedic medicine. Broken bones carried many meanings in relation to our thinking through the meeting places of medical sociology, disability studies and sociology of disability, and the fractures that remain between these disciplines. The immediacy of a broken bone recalled the efforts in the broad and contested field of disability studies to center the body/mind and bring impairment into the frame alongside its mobilization against ableism, within and beyond the academy. Bones are referred to in “Skin, tooth and bone,” the disability justice primer written by the foundational group (Sins Invalid, 2019), signaling the centrality of social movements as backbones and visionaries of crip futures. This image of the bone also alludes to “breaking open the bone” of mad grief and the growing presence of Mad studies as its own emerging field (Poole and Ward, 2013; Willer et al., 2021). Lastly, in looking close-up at a fractured bone, we are reminded of the corporeality that connects us and the importance of scale and perspective for framing our understanding of social phenomena.

This Research Topic is woven together across many points of convergence, though three themes stand out: (1) disabled world making, (2) communities of care for longstanding wellbeing, and (3) novel research methods. By disabled world making, we mean how disabled people make and remake their worlds: in arts, cultural practices, through activism, and more. Many of these studies highlighted the skills, knowledge-practices and resilience of disabled people. Bringing many theories into dialogue, da Silva et al. challenge reductionist ideas of disability. They propose a complexity paradigm to understand disability as characteristic of human diversity, rather than deviation or pathology. Landry documents mad people's world making, specifically consumer/survivor led businesses that were created in the 1980s and 1990s to counter their exclusion from the mainstream labor market. Her work accounts for these small businesses as significant sites of mad people's advocacy in Ontario, where activist knowledge-practices were fostered through community organizing. Padilla and Tan present us with an innovative decolonial methodology that disrupts disciplinary boundaries and offer two gnosis-based, embodied counter-stories. These stories defy traditional epistemologies and embrace the diverse forms of knowledge and emotional production that disability can generate. Soldatic et al. employ participatory approaches, including the co-creation of AI-generated e-books, to study the important role of everyday technologies among culturally and linguistically diverse (CaLD) migrants with disabilities in remaking their worlds as new communities. The study highlights not only the barriers and burden CaLD migrants with disabilities experience but also, emphasizes participants' agency and creativity in navigating digital spaces.

The second thread, communities of care for longstanding wellbeing, refers to a whole host of community practices of wellbeing and care. Research within these texts amplified lessons from crip and mad kinship on keeping each other well. Here interdependence is both a reality and an aspiration. We found commonality across research that distilled shared and mutually constitutive practices of wellbeing, generating new insights and potential alliances that are necessary to build, mobilize, and sustain crosscutting communities of wellbeing. Ellis et al. write transparently about the first year of their 5-year co-produced research study, *Cripping Breath*, and the care-full work they undertake to ensure their research practices reflect the ethics and purpose of the project. This care extends to think through crip time, embrace slow scholarship, compensate community researchers and talk explicitly about grief, loss, and legacy in research processes. From a caregiver perspective, Ke shares lessons drawn from her experience of caring for her sister who has critical brain injuries. Ke uses a phenomenological approach to push back against ableist ideas of disability as deficit or a thing to overcome, and instead supports her sister's recovery by attuning to their current lived reality, to honor the changed condition of their body/mind. Middelmann's reflections on the connections between ethics, methods and values in public space research over several years in Johannesburg lead him to conclude that reciprocal practices of wellbeing require internal shifts toward others as well as interdependence and collaboration across difference. Lastly, Yepthomi et al. introduce us to Indigenous approaches to mental distress among northeast Indian Naga communities, arguing Indigenous epistemologies recognize healing as a collective process.

Lastly, and in speaking most directly to the Research Topic's central call, several articles recounted novel sociological research methodologies and methods. Rooted in disability, crip and mad research praxis, they emphasize the importance of a strong commitment to accessibility that supports meaningful engagement and knowledge co-production with disability communities. Taking up and taking in disability theory in research practices and community engagement, as these authors suggest, requires creativity, shifting temporalities and technological innovation. For example, Beesley revisits the crucial role of Emancipatory Disability Research (EDR), critically analyzing its impact, possibilities and features that remain necessary for an anti-ableist praxis, while expanding its canon. What should be preserved from EDR, he argues, is an emphasis on “*empowering subjects and its democratization of research practice*.” Ryan centers joy in disability research and highlights the disruptive potential of bringing a crip “joyful” approach to narrative research. Narrative portraiture is presented as a participant-centered method that can produce nuanced counternarratives of siblinghood and disability. Sinclair thinks with Mad Time and its potential to disrupt normative and sanist research practices. She points to the violence produced by conventional methodologies which reproduce psychiatric relations and proposes the generative opportunities of Mad Time to be a subversive alternative approach. Wechuli considers what it means to crip ethnographic research as an emancipatory reorientation, including autoethnography and its subgenre evocative autoethnography. Wechuli's work aligns with others in the Research Topic, in terms of affective relations of crip time and resistance to disablism, ableism, and sanism in academia. Though we center these three threads across projects, we invite you to locate other points of connection and contention, as you make your way through this Research Topic of empirical and analytic papers.
